# ASSOCIATION OF HEALTHCARE USE AND EXPENDITURE BASED ON DUAL ELIGIBILITY AND ENROLLMENT IN MANAGED CARE

**DOI:** 10.1093/geroni/igae098.4387

**Published:** 2024-12-31

**Authors:** Mohammad Usama Toseef, Aastha Dharia, Ramin Homayouni, Wassim Tarraf

**Affiliations:** Corewell Health, Royal Oak, Michigan, United States; Oakland University William Beaumont School of Medicine, Rochester, Michigan, United States; Oakland University William Beaumont School of Medicine, Rochester, Michigan, United States; Wayne State University, Detroit, Michigan, United States

## Abstract

Individuals enrolled in both Medicaid and Medicare, also known as duals, have complex and high healthcare needs and disproportionately account for higher state and federal spending. In recent years, most of these beneficiaries have been enrolled in some form of managed care plans. In this study, using the 2020-2021 Medical Expenditure Panel Survey, we evaluate the association of dual-eligibility status and different forms of healthcare use and expenditures: inpatient, emergency room and outpatient. We also explore if enrollment in a managed care affects this relationship. We estimate a series of Poisson regressions for use variables and generalized linear models for expenditure variables that account for the interaction between dual-eligibility status and enrollment in managed care. Postestimation, we calculate marginal effects and use analysis of variance contrast tests to estimate the differences in the predicted use events and average expenditure based on managed care and dual-eligibility status. We find that, on average, duals eligibles have a higher use of inpatient and emergency room than non-duals but similar outpatient visits. We also find that non-duals enrolled in managed care have lower outpatient and emergency room visits than their non-managed care counterparts. No significant differences are observed for dual eligibles in terms of their managed care status for both use and expenditures. While more research and years of data are needed to establish whether managed care improves utilization and lowers spending for dual eligibles, we find that managed care affects duals and non-duals differently and stakeholders should consider this to provide equitable health.

